# Supplementary material for: Alcohol consumption in the general population is associated with structural changes in multiple organ systems
Source: eLife. 2021 Jun 1;10:e65325. doi: 10.7554/eLife.65325 (PMC8192119; doi:10.7554/eLife.65325)
Supplement: Supplementary file 1. [file elife-65325-supp1.docx]

**Supplementary Table 1. Alcohol consumption (g/d) in all and males and females for brain grey matter (N=10143), brain white matter (N=9053), heart (N=11821) aorta (N=12376) and liver (N=3649).**

| **Alcohol (g/d)** | **Brain grey matter** | **Brain white matter** | **Heart** | **Aorta** | **Liver** |
| --- | --- | --- | --- | --- | --- |
| **All (median)** | 14.29 | 14.29 | 14.29 | 14.29 | 16.61 |
| **IQR** | 6.46-26.78 | 6.26-26.79 | 6.70-26.79 | 6.69-26.79 | 8.93-28.86 |
| **Males (median)** | 20.94 | 20.97 | 21.43 | 20.99 | 23.52 |
| **IQR** | 10.28-35.78 | 10.27-35.75 | 10.71-36.60 | 10.28-36.19 | 12.93-38.79 |
| **Females (median)** | 10.71 | 10.71 | 10.71 | 10.71 | 12.47 |
| **IQR** | 3.57-17.89 | 3.57-17.86 | 3.99-18.58 | 3.89-17.31 | 6.80-21.43 |

IQR: Interquartile range
