# Supplementary material for: Alcohol consumption in the general population is associated with structural changes in multiple organ systems
Source: eLife. 2021 Jun 1;10:e65325. doi: 10.7554/eLife.65325 (PMC8192119; doi:10.7554/eLife.65325)
Supplement: Supplementary file 2. [file elife-65325-supp2.docx]

**Supplementary Table 2. Coefficients for log_2_ alcohol and age in an expanded model for cardiac (N=11,821) and aortic (N=12,376) imaging phenotypes including tests of interactions between log_2_ alcohol and age.**

|  | **Alcohol** | **Alcohol x Age** | **Age** |
| --- | --- | --- | --- |
| **Heart** | ***Beta, P-value*** | ***Beta, P-value*** | ***Beta, P-value*** |
| Left ventricular mass index | 1.84, 3.2×10^-10^ | -0.02, 3.5×10^-7^ | -0.01, 0.42 |
| Left ventricular end-diastolic volume index | 2.25, 6.9×10^-5^ | -0.03, 3.5×10^-3^ | -0.24, 3.4×10^-11^ |
| Left ventricular ejection fraction (%) | 0.17, 0.50 | -0.002, 0.62 | 0.04, 6.8×10^-3^ |
| Right ventricular end-diastolic volume index (ml/m^2^) | 1.99, 1.0×10^-3^ | -0.02,0.018 | -0.25, 4.7×10^-11^ |
| Right ventricular ejection fraction (%) | -0.04, 0.87 | 0.001, 0.72 | 0.04, 0.024 |
| Left atrial volume index (ml/m^2^) | 0.59, 0.23 | -0.002, 0.75 | -0.11, 4.4×10^-4^ |
| Right atrial volume index (ml/m^2^) | 0.25, 0.65 | 1.4x10^-4^, 0.99 | 0.04, 0.22 |
| **Aorta** |  |  |  |
| Ascending aortic area index (mm^2^/m^2^) | 7.4, 0.06 | -0.08, 0.22 | 3.0, 1.5×10^-34^ |
| Descending aortic area index (mm^2^/m^2^) | 5.9, 6.9×10^-4^ | -0.07, 8.1×10^-3^ | 2.1, 2.9×10^-83^ |
| Ascending aortic distensibility (%/mmHg) | -0.21, 1.4×10^-7^ | 0.003, 2.6×10^-7^ | -0.1, <1.0×10^-300^ |
| Descending aortic distensibility (%/mmHg) | -0.20, 4.3×10^-6^ | 0.003, 2.7×10^-5^ | -0.10, 6.0×10^-293^ |

Regression model: Imaging phenotype=log_2_ Alchohol + log_2_Alcohol* Age + Age + Sex + Ethnicity + BMI + Hypertension + Diabetes + Ever smoked + College degree
